# ‘*Candidatus* Rickettsia asemboensis’ and *Wolbachia* spp. in *Ctenocephalides felis* and *Pulex irritans* fleas removed from dogs in Ecuador

**DOI:** 10.1186/s13071-014-0455-0

**Published:** 2014-09-30

**Authors:** José A Oteo, Aránzazu Portillo, Francisco Portero, Jorge Zavala-Castro, José M Venzal, Marcelo B Labruna

**Affiliations:** Departamento de Enfermedades Infecciosas, Hospital San Pedro-Centro de Investigación Biomédica de La Rioja (CIBIR), C/ Piqueras 98, 26006 Logroño (La Rioja), Spain; Escuela Superior Politécnica de Chimborazo, Riobamba, Ecuador; Universidad Autónoma de Yucatán. Centro de investigaciones regionales “Dr. Hideyo Noguchi”, Mérida, Yucatán Mexico; Laboratorio de Vectores y Enfermedades Transmitidas and Departamento de Parasitología Veterinaria, Facultad de Veterinaria, Universidad de la República, Regional Norte, Salto, Uruguay; Departamento de Medicina Veterinária Preventiva e Saúde Animal, Faculdade de Medicina Veterinária e Zootecnia, Universidade de São Paulo, São Paulo, Brazil

**Keywords:** Fleas, *Pulex irritans*, *Ctenocephalides felis*, Ecuador, ‘*Candidatus* Rickettsia asemboensis’, *Wolbachia* spp., *Bartonella* spp., *Yersinia pestis*, Plague

## Abstract

**Background:**

Flea-borne infections are distributed worldwide. Up to date there are no reports about microorganisms associated to fleas in Ecuador.

**Methods:**

Seventy-one *Pulex irritans* and 8 *Ctenocephalides felis* fleas were removed from dogs in two Ecuadorian areas (Pastaza and Chimborazo Provinces) in December 2012. DNA extracts were tested by polymerase chain reaction (PCR) assays targeting universal 16S rRNA, as well as screened for the presence of *Rickettsia* spp. (*gltA*, *htrA*, *ompB, sca4* and *ompA* genes) and *Bartonella* spp. (*rpoB, gltA* and ITS genes).

**Results:**

Our results showed the presence of ‘*Candidatus* Rickettsia asemboensis’ (highly similar to *R. felis*) in *C. felis* and *Wolbachia* spp. endosimbionts in *P. irritans* collected from animals in Ecuador. No fleas were found to be positive for any *Bartonella* species or *Yersinia pestis*.

**Conclusions:**

Clinicians should be aware of the potential risk of this new *Candidatus* Rickettsia sp. and keep in mind other flea-borne infections since these flea species frequently bite humans.

## Background

Flea-borne diseases are worldwide-distributed emerging and re-emerging infections. Among them, plague, which is caused by *Yersinia pestis*, is the most severe human infection transmitted by fleas [1]. In South America, permanent plague foci exist among native rodent and flea populations in Bolivia, Brazil, Ecuador and Peru [2]. Rats have been the responsible hosts and from them, the disease has spread to other rodents. Ecuador is considered a plague ‘hot-spot’ since its introduction in 1908, and has experienced important outbreaks. Chimborazo Province has historically been a highly endemic area and the last fatal Ecuadorian cases of plague were reported there in 2004 [3,4].

In addition, fleas are vectors of murine typhus (caused by *Rickettsia typhi*), flea-borne spotted fever (caused by *Rickettsia felis*) and harbour *Bartonella* spp. [1,5-7]. Recent evidence of murine typhus in Ecuador is lacking, but the disease may be endemic in localities where commensal rodents (*Rattus* spp.) are abundant. To the best of our knowledge, data about distribution of *R. felis* in this country are unknown and there are no reports describing *Bartonella* spp. in fleas from Ecuador. For these reasons, our interest was focused on the study of flea-borne agents in two Ecuadorian areas (one of them where the last plague outbreak occurred) using molecular biological methods [polymerase chain reaction (PCR) and DNA sequencing].

## Methods

In December 2012, a total of 79 fleas were removed from dogs by members of Red Iberoamericana para la Investigación y Control de las Enfermedades Rickettsiales, Programa Iberoamericano de Ciencia y Tecnologías para el Desarrollo (RIICER, CYTED; no. 210RT0403). Fifty-two specimens (44 *Pulex irritans* and 8 *Ctenocephalides felis*) were collected in the Estación Experimental Fátima (ESPOCH), Cantón Puyo, in Pastaza Province (01°24’34.6”S; 77°59’57.5”W), and 27 specimens (all *P. irritans*) were collected in Cantón Guamote, in Chimborazo Province (02°00’25.0”S; 78°47’09.3”W). The study areas had altitudes of 1,034 m and 3,657 m, respectively. After identification at the species level, samples were kept in 70% ethanol at room temperature before being tested. DNA of each arthropod was extracted by lysis with 0.7 M ammonium hydroxide and tested by PCR with the universal primers fD1 and rp2 [8]. This primer pair amplifies the main part of the 16S rRNA gene and has been used for the identification of *Y. pestis* as the causative agent of plague in India [9]. Samples were also screened for the presence of *Rickettsia* spp. with PCR assays targeting rickettsial citrate synthase (*gltA*) and 17 kDa antigen (*htrA*) genes [10,11]. In accordance with the taxonomic scheme [12], additional rickettsial genes (*ompB, sca4* and *ompA*) were tested to properly identify *Rickettsia*-positive specimens [13-17]. Moreover, *Bartonella* spp. was tested using RNA polymerase β-subunit–encoding gene (*rpoB*), *gltA* and intergenic spacer region gene (ITS) PCR primers, which amplify fragments of *Bartonella* genes [18-20].

Each PCR included positive controls consisting of *Bartonella henselae* DNA extracted from a cat flea (*C. felis*) from La Rioja - Spain, or *Rickettsia slovaca* strain S14ab DNA (obtained from Vero cells inoculated in our facility with a *Dermacentor marginatus* tick from La Rioja - Spain, and known to be infected with *R. slovaca*). Negative controls (DNA-free water) were included in all assays. Sequences generated by each pair of primers were then compared with those in GenBank using BLAST (www.ncbi.nlm.nih.gov/blast/Blast.cgi).

## Results and discussion

PCR assays using universal eubacterial primers for 16S rRNA gene yielded amplicons of different intensity for 69 out of 79 fleas (8/8 *C. felis* and 35/44 *P. irritans* from Pastaza and 26/27 *P. irritans* from Chimborazo). All *C. felis* (n = 8) were also found to be infected with *Rickettsia* species using *gltA* and *htrA* as rickettsial PCR targets, whereas no evidence of *Rickettsia* spp. was found in *P. irritans*. Moreover, no sample was positive for *Bartonella* species as determined either by *rpoB*, *gltA* or ITS PCR assays. Positive and negative controls worked as expected in all cases.

Sequences of rickettsial 16S rRNA gene obtained from 7/8 *C. felis* (1303–1373 bp) showed the closest identity (99.6-99.9%) with ‘*Candidatus* Rickettsia asemboensis’, a potentially new *Rickettsia* species according to the established criteria [12,21] (Table 1). In these samples, the percentage of identity with 16S rRNA gene of a validly published *Rickettsia* species reached 99.4% for *R. felis* (accession no. NR074483). The 1387 bp-long sequence of 16S rRNA gene obtained from the remaining *C. felis* had the highest identity (97.1%) with a sequence from uncultured flea-associated bacterium [22], and showed 93.6% identity with *Snodgrasella alvi,* betaproteobacteria classified in the family *Neisseriaceae* and previously isolated from the bee gut [23] (Table 1).

**Table 1:** **Results of nucleotide sequence analysis corresponding to PCR products amplified from the 8** ***Ctenocephalides felis*** **specimens of this study**

Gene*	**Flea gender** ^**1**^	**Location**	**Length of sequence (bp)**	**% identity**	**Bacteria with closest identity in the BLAST search**	**GenBank accession no.**
16S rRNA	1/2 M; 6/6 F	Pastaza	1303-1373	99.6-99.9	*‘Candidatus* Rickettsia asemboensis’ (*Rickettsia* F30)	JN315967
	1/2 M; 0/6 F	Pastaza	1387	97.1	Uncultured flea-associated bacterium	EU137419
				93.6	*Snodgrasella alvi*	JQ746645
*gltA*	2/2 M; 6/6 F	Pastaza	350	100	*Rickettsia felis*-like (*Rickettsia* RF2125)	AF516333
				99.7	*‘Candidatus* Rickettsia asemboensis’ (*Rickettsia* F30)	JN315968
*htrA*	2/2 M; 6/6 F	Pastaza	394	100	*‘Candidatus* Rickettsia asemboensis’ (*Rickettsia* F30)/*Rickettsia felis*-like (*Rickettsia* SE313/cf1and5)	JN315969/DQ166937/AY953286
*ompB*	2/2 M; 6/6 F	Pastaza	464	100	*‘Candidatus* Rickettsia asemboensis’ (*Rickettsia* F30)	JN315972
*sca4*	2/2 M; 6/6 F	Pastaza	352	100	*‘Candidatus* Rickettsia asemboensis’ (*Rickettsia* F30)	JN315970

*Inconclusive sequences were obtained for *ompA* gene.

^1^Number of positive specimens / Total number of specimens from each flea gender; M: Male; F: Female.

Sequencing of *gltA* fragments (350 bp) identified *R. felis*-like rickettsiaceae in *C. felis* (100% identity with *Rickettsia* sp. genotype RF2125) [24] and showed 99.7% identity (349/350 bp) with ‘*Candidatus* R. asemboensis’ (Table 1). The sequences of the 17-kDa amplicons (394 bp) were homologous (100% identical) to each other and to ‘*Ca.* R. asemboensis’ as well as to other molecular isolates included in the *R. felis*-like genotype group (*Rickettsia* sp. SE313 detected in *Echidnophaga gallinacea* from Egypt and *Rickettsia* sp. cf1and5 detected in *C. felis* from USA) (Table 1).

The sequences of *ompB* and *sca4* amplicons (464 and 352 bp, respectively) were also 100% identical to ‘*Ca.* R. asemboensis’ (Table 1). Unfortunately, *ompA* PCR primers did not yield amplicons of the expected size, and inconclusive sequences were obtained for this target gene.

Percentages of identity with validated species *R. felis* (accession no. CP000053) were 98.6, 96.2, 97.9 and 96.6% for *gltA*, *htrA*, *ompB* and *sca4* genes, respectively.

In addition, 59 out of 61 sequences of 16S rRNA gene obtained from 61 *P. irritans* specimens provided evidence of the presence of probable endosymbionts similar to those found within other arthropods and belonging to the genus *Wolbachia* (Table 2). Unfortunately, in two cases (corresponding to two *P. irritans* samples from Chimborazo) it was not possible to get a good-quality sequence to identify the bacteria.

**Table 2:** **Results of nucleotide sequence analysis corresponding to 59 PCR products amplified** ^**1**^ **from the 71** ***Pulex irritans*** **of this study (44 specimens from Pastaza and 27 from Chimborazo)**

**Gene**	**Flea gender** ^**2**^	**Location**	**Length of sequence (bp)**	**% identity**	**Bacteria with closest identity in the BLAST search**	**Genbank**
16S rRNA	9/16 M; 10/28 F	Pastaza	1287-1386	98.3-100	*Wolbachia* sp. wRi endosymbiont of *Drosophila simulans*	NR074437
	11/13 M; 13/14 F	Chimborazo				
	4/16 M; 2/28 F	Pastaza	1338-1383	98.4-98.7	*Wolbachia* sp. endosymbiont of *Pseudolynchia canariensis*	DQ115538
	0/16 M; 2/28 F	Pastaza	1300-1372	98.3-99.3	*Wolbachia* sp. endosymbiont of *Gryllus crickets*	U83094
	0/16 M; 1/28 F	Pastaza	1305	99.3	*Wolbachia* sp. endosymbiont of *Gryllus ovisopis*	U83093
	0/16 M; 1/28 F	Pastaza	1374	98.3	*Wolbachia* sp. endosymbiont of *Curculio hachijoensis*	AB746399
	0/16 M; 6/28 F	Pastaza	1291-1368	98.3-99	*Wolbachia* sp. endosymbiont of *Kleidocerys resedae*	JQ726770

^1^In two cases we did not obtain enough good-quality sequences to identify the bacteria, and we did not obtain amplicons for ten specimens.

^2^Number of positive specimens / Total number of specimens from each flea gender; M: Male; F: Female.

In our study ‘*Candidatus* R. asemboensis’ has been found in fleas that bite humans (*C. felis*) removed from dogs in Ecuador. This potential new species was previously detected in *Ctenocephalides canis* from Kenya and whether it is a human pathogen remains unknown [21]. ‘*Ca.* R. asemboensis’ is highly similar to *Rickettsia* RF2125, a member of the *R. felis*-like genotype group that circulates in fleas from Uruguay [25]. Apart from epidemic typhus (caused by *Rickettsia prowazekii* and transmitted by lice), no data about human diseases associated with *Rickettsia* species have been published from Ecuador [26]. Nevertheless, in the Pastaza province (one of our sampling areas) cases of acute undifferentiated febrile illness compatible with rickettsioses have been reported [27].

Up to the present study, the presence of *R. felis* and/or *Bartonella* spp. has not been demonstrated in fleas from Ecuador. However, *R. felis* has been found in South American fleas from Brazil, Peru, Uruguay, Chile, Argentina and Colombia [11,25,28-31]. In addition, human infection with *R. felis* in South America has been confirmed in Brazil by molecular methods [32], and human serological evidence of *R. felis* infection has been recently reported in Colombia [33]. Moreover, there are limited reports describing *Bartonella* spp. in fleas from South America. A molecular study conducted in a *Pulex* specimen found on a Peruvian person evidenced the presence of a potential new *Bartonella* species [5]. Years later, our research group detected *B. rochalimae*, *B. clarridgeiae*, and *B. henselae* in *P. irritans* and *C. felis* collected from cats and dogs in Chile, suggesting the role of fleas as possible vectors of *Bartonella* spp. [7]

Lastly, *Wolbachia* spp. are alphaproteobacteria included in the family *Anaplasmataceae* that were first detected in fleas in 2000 [34]. In this study, the detection rate of *Wolbachia* spp. in *P. irritans* was 83% (59/71). On the contrary, no *C. felis* analysed (0/8) showed evidence of carriage of *Wolbachia* endosymbionts. Previous studies had identified *Wolbachia* in around 20% of cat fleas [35,36]. It has been suggested that *R. felis* infection in fleas might diminish the richness of flea microbiota [37]. According to our data, there is a strong association of *C. felis* with ‘*Ca.* R. asemboensis’ whereas *P. irritans* is associated with *Wolbachia* spp. Nevertheless, the interaction of *Wolbachia* with *R. felis* or other related species, such as ‘*Ca.* R. asemboensis’ in fleas needs further investigation.

Based on 16S rDNA analysis, the presence of *Y. pestis* DNA has not been demonstrated in our fleas despite *P. irritans* having been previously described as vectors of *Y. pestis* in Ecuador [2] and plague outbreaks have been repeatedly reported in the Chimborazo region [3,4], where some flea specimens were collected. The rodent flea *Xenopsilla cheopis*, which is the main vector, does not exist in the inter-Andean region of Ecuador (2,500-4,000 m above sea level) possibly, due to very sudden changes in the climatic conditions [38].

## Conclusions

In summary, our result confirms the presence of ‘*Candidatus* R. asemboensis’ and *Wolbachia* spp. in fleas removed from dogs in Ecuador. Clinicians should be aware of the potential risk of this new *Candidatus* Rickettsia sp. and keep in mind other flea-borne infections in areas where humans are exposed to fleas.
